# Prediction of extubation outcome: a randomised, controlled trial with automatic tube compensation vs. pressure support ventilation

**DOI:** 10.1186/cc7724

**Published:** 2009-02-23

**Authors:** Jonathan Cohen, Maury Shapiro, Elad Grozovski, Ben Fox, Shaul Lev, Pierre Singer

**Affiliations:** 1 General Intensive Care Unit, Rabin Medical Center, Beilinson Campus, Petah Tikva, 49100, Israel

## Abstract

**Introduction:**

Tolerance of a spontaneous breathing trial is an evidence-based strategy to predict successful weaning from mechanical ventilation. Some patients may not tolerate the trial because of the respiratory load imposed by the endotracheal tube, so varying levels of respiratory support are widely used during the trial. Automatic tube compensation (ATC), specifically developed to overcome the imposed work of breathing because of artificial airways, appears ideally suited for the weaning process. We further evaluated the use of ATC in this setting.

**Methods:**

In a prospective study, patients who had received mechanical ventilation for more than 24 hours and met defined criteria for a weaning trial, underwent a one-hour spontaneous breathing trial with either ATC (n = 87) or pressure support ventilation (PSV; n = 93). Those tolerating the trial were immediately extubated. The primary outcome measure was the ability to maintain spontaneous, unassisted breathing for more than 48 hours after extubation. In addition, we measured the frequency/tidal volume ratio (f/VT) both with (ATC-assisted) and without ATC (unassisted-f/VT) at the start of the breathing trial as a pretrial predictor of extubation outcome.

**Results:**

There were no significant differences in any of the baseline characteristics between the two groups apart from a significantly higher Acute Physiology and Chronic Health Evaluation (APACHE) II score in the ATC group (p = 0.009). In the PSV group, 13 of 93 (14%) patients failed the breathing trial compared with only 6 of 87 (6%) in the ATC group; this observed 8% difference, however, did not reach statistical significance (p = 0.12). The rate of reintubation was not different between the groups (total group = 17.3%; ATC = 18.4% vs. PSV = 12.9%, p = 0.43). The percentage of patients who remained extubated for more than 48 hours was similar in both groups (ATC = 74.7% vs. PSV = 73.1%; p = 0.81). This represented a positive predictive value for PSV of 0.85 and ATC of 0.80 (p = 0.87). Finally, the ATC-assisted f/VT was found to have a significant contribution in predicting successful liberation and extubation compared with the non-significant contribution of the unassisted f/VT (unassisted f/VT, p = 0.19; ATC-assisted f/VT, p = 0.005).

**Conclusions:**

This study confirms the usefulness of ATC during the weaning process, being at least as effective as PSV in predicting successful extubation outcome and significantly improving the predictive value of the f/VT.

**Trial registration:**

Current Controlled Trials ISRCTN16080446

## Introduction

Successful weaning and liberation from mechanical ventilation remain critical stages of a patient's intensive care unit (ICU) stay. Tolerance of a spontaneous breathing trial is an evidence-based strategy to predict successful weaning from mechanical ventilation [1]. These trials have traditionally been performed while the patient receives varying levels of ventilatory support, including, in recent studies, continuous positive airway pressure (CPAP) [2], a T-tube circuit [3] or low-level pressure support ventilation (PSV) [4]. The level of support may be relevant to whether the breathing trial is tolerated, because it has been argued that, for some patients, weaning failure may be attributable to the respiratory load imposed by the endotracheal tube [5]. In support of this, Koksal and colleagues have demonstrated a significant increase in the endocrine stress response during a breathing trial [6]. The magnitude of the response was influenced by the mode used, being significantly greater at the end of a breathing trial with a T-tube than with either PSV or CPAP.

Automatic tube compensation (ATC) has been developed to overcome the imposed work of breathing due to artificial airways [7]. It delivers the exact amount of pressure necessary to overcome the resistive load of the endotracheal tube for the flow measured at the time, without affecting the patient's breathing pattern [8]. It potentially simulates spontaneous breathing without the endotracheal tube, so it has been designated as 'electronic extubation' [7]. This mode of ventilation thus seems ideally suited for use during the weaning period.

PSV was widely used in the performance of a spontaneous breathing trial and has been shown to compensate for the additional work of breathing imposed by the endotracheal tube [9]. However, studies have shown that compared with PSV, ATC was more effective in overcoming the work of breathing necessary to overcome endotracheal tube resistance [10]. ATC was also perceived to be more comfortable by normal volunteers [11] and resulted in less ineffective ventilator-triggering as a result of auto-positive end expiratory pressure (PEEP) [7]. However, the ventilator used in all these studies was equipped with prototype ATC software, not available in commercial mechanical ventilators.

It was the aim of the present prospective study to further assess the value of ATC in predicting successful weaning. To do this, we assessed extubation outcome after a spontaneous breathing trial with ATC and compared it with PSV. Additionally, we assessed whether the predictive value of the frequency to tidal volume ratio (f/VT), widely used for predicting successful extubation, could be enhanced by the addition of ATC, that is, ATC-assisted f/VT.

## Materials and methods

### Patients

This prospective, randomised, controlled trial was approved by the local Institutional Review Board and performed in the 12-bed general ICU of Rabin Medical Center between October 2006 and April 2008. Patients were eligible for enrolment if they met the following criteria: required mechanical ventilation for more than 24 hours and considered ready for weaning. Criteria of readiness for weaning included all the following: improvement of the cause of respiratory failure; oxygen saturation of 92% or higher with a fraction of inspired oxygen (FiO_2_) of 50% or less; stable neurological status (Glasgow Coma Score > 8); require bronchial toilet less than twice in the eight hours preceding the assessment; no need for vasoactive drugs; receiving only minimal or no sedation; body temperature between 36 and 38°C; and level of pressure support of 15 cmH_2_O or less with a PEEP level of 8 cmH_2_O or less.

### Measures

The following parameters were recorded before performing the spontaneous breathing trial: demographic data, including age, sex, admission diagnosis, admission Acute Physiology and Chronic Health Evaluation (APACHE) II score [12], duration of mechanical ventilation and length of ICU stay; haemodynamic data, including heart rate and mean arterial blood pressure; fluid balance in the 24 hours preceding the start of the spontaneous breathing trial; and ventilatory data, including level of PEEP, tidal volume, partial carbon dioxide tension in arterial blood (paCO_2_), respiratory rate, minute ventilation, ratio of partial oxygen tension in arterial blood to fraction of inspired oxygen (PaO_2_/FiO_2 _ratio) and f/VT (breaths/minute/L). The f/VT, assessed first with and immediately thereafter without 100% ATC, was calculated in both groups after one minute of spontaneous breathing with PEEP of 5 cmH_2_O and no mandatory machine breaths supplied from the ventilator [13]. Values were displayed on the ventilator and the value used was the average of three breaths. At the time of measurement, patients were ventilated with PSV (level 9.2 ± 1.3 cmH_2_O; mean ± standard deviation), FiO_2 _less than 0.5 (mean level 0.38 ± 0.01) and PEEP of 5 cmH_2_O. Ventilation was performed with either the Evita-4 ventilator (Drager, Lubeck, Germany; n = 117) or the Puritan-Bennett 840 ventilator (Puritan-Bennett Corporation, CA, USA; n = 63) depending on the equipment assigned to each patient bed.

### Procedures

After meeting inclusion criteria, informed consent was obtained from the patient or surrogate decision maker. Patients were then randomly assigned, in a blinded fashion with the use of opaque, sealed envelopes, to undergo a one-hour spontaneous breathing trial with either ATC (patients breathed through the ventilatory circuit using flow-triggering and CPAP of 5 cmH_2_O, FiO_2 _less than 0.5 with the addition of ATC 100%; the ATC group) or PSV (patients breathed through the ventilatory circuit using flow-triggering and CPAP of 5 cmH_2_O, FiO_2 _less than 0.5 with the addition of 7 cmH_2_O of pressure support; the PSV group). These parameters were maintained throughout the trial. Tolerance of the trial was continuously evaluated. Features of poor tolerance included: respiratory rate above 35 breaths/minute for five minutes or longer, arterial saturation less than 90%, increase in heart rate above 140 beats/minute, increase in systolic blood pressure above 180 mmHg or decrease to less than 90 mmHg, and increased anxiety, diaphoresis or thoraco-abdominal paradox. For patients not tolerating the breathing trial, full ventilatory support was reinstituted, while patients who tolerated the trial underwent immediate extubation and received supplemental oxygen via a face mask.

Following extubation, reintubation was performed in the following conditions: hypoxaemia (oxygen saturation below 92% for more than five minutes while receiving FiO_2 _more than 0.5); presence of respiratory acidosis (arterial pH below 7.35 with paCO_2 _above 45 mmHg); inability to protect the airway because of upper airway obstruction (stridor); and evidence of excessive respiratory work (respiratory rate 35 breaths/minute of above for more than five minutes, diaphoresis or thoraco-abdominal paradox). The reason for and time to reintubation (rounded off to the nearest hour) were noted. The spontaneous breathing trial was performed by two investigators (JC and MS). Decisions regarding reintubation were made by caregivers who were blinded to the treatment group.

### Outcome variables

The primary outcome measure was successful extubation, defined as the ability to maintain spontaneous, unassisted breathing for longer than 48 hours after removal of the endotracheal tube. This definition encompasses both the number of patients tolerating the breathing trial and the number able to maintain spontaneous breathing after extubation. In addition we assessed the value of the ATC-assisted f/VT as compared with the unassisted-f/VT as a predictor of successful extubation.

### Statistical analyses

Differences between the ATC and PSV groups in baseline, respiratory and haemodynamic characteristics were analysed using Student's *t*-test for independent samples (for continuous variables) and differences between groups in patient course and outcome were analysed using the chi-squared test (for categorical variables). Positive predictive values for PSV and ATC with successful extubation without reintubation as the outcome end-point were also calculated. Prediction of extubation by pretest f/VT was examined using the Student's *t*-test. Receiver operating curves (ROC) were constructed for determining the prognostic accuracy of the ATC-assisted f/VT in predicting successful liberation and extubation. In addition, patients were divided into categories according to f/VT using arbitrary steps of 25 breaths/minute/L. Statistical analyses were performed using the statistical software SPSS 15.0 for Windows (Chicago, IL, USA). Statistical results were considered significant at p < 0.05.

## Results

### Patient enrollment and demographics

Of the 180 patients included in the study, 87 were randomised to the ATC group and 93 to the PSV group. Baseline characteristics are shown in Table 1. There were no significant differences at baseline between the two groups regarding patient characteristics and indication for mechanical ventilation, apart from a significantly higher APACHE II score in the ATC group (p = 0.009).

**Table 1:** Baseline characteristics by group at start of spontaneous breathing trial

**Characteristic**	**ATC group** **(n = 87)**	**PSV group** **(n = 93)**	**p value**
Age, years	62.1 ± 16.6	66.1 ± 18.1	0.13
Sex (male : female)	55:32	60:33	0.86
Days ventilated before SBT percentage ventilated	5.9 ± 3.5	6.3 ± 4.7	0.56
> 8 days before SBT	18.3	23	0.61
Causes of ARF			0.15
COPD exacerbation, n (%)	7 (8.1)	11 (11.8)	
Pneumonia, n (%)	17 (19.5)	19 (20.5)	
Sepsis with ALI, n (%)	20 (22.9)	18 (19.3)	
Multi-trauma, n (%)	10 (11.5)	13 (13.9)	
Post-operative, n (%)	14 (16.1)	12 (12.9)	
Heart failure, n (%)	12 (13.7)	8 (8.6)	
Other, n (%)	7 (8.2)	12 (12.9)	
APACHE II score	22.1 ± 7.9	19.1 ± 7.0	0.009
Endotracheal tube size, mm	7.8 ± 0.4	7.8 ± 0.4	0.73

Continuous data are presented as mean ± standard deviation. Binary data are presented as n (percentage).

ALI = acute lung injury; APACHE II = Acute Physiology and Chronic Health Evaluation II severity of illness; ARF = acute respiratory failure; ATC = automatic tube compensation; COPD = chronic obstructive pulmonary disease; PSV = pressure support ventilation; SBT = spontaneous breathing trial.

### Respiratory and haemodynamic characteristics at the start of the spontaneous breathing trial

These are shown in Table 2. There were no significant differences between the ATC and PSV groups in any of the respiratory or haemodynamic parameters studied.

**Table 2:** Respiratory and haemodynamic characteristics by group at start of spontaneous breathing trial

**Characteristic**	**ATC** **(n = 87)**	**PSV** **(n = 93)**	**p value**
Heart rate, beats/minute	91.5 ± 1.9	84.2 ± 1.9	0.85
MAP, mmHg	93.2 ± 1.8	91.9 ± 1.8	0.63
PaCO_2_, mmHg	43.5 ± 1.3	42.8 ± 1.2	0.60
PaO_2_/FiO_2 _ratio	269.8 ± 10.3	271.7 ± 10.8	0.79
Respiratory rate, breaths/minute	20.5 ± 0.7	20.5 ± 0.7	0.81
Tidal volume, ml	0.48 ± 0.1	0.47 ± 0.1	0.78
Minute ventilation, L/minute	8.7 ± 0.5	9.2 ± 0.4	0.40
Fluid balance 24 hours before SBT, ml	-48. 9 ± 1322.0	36.2 ± 1282.4	0.67

Data are presented as mean ± standard deviation.

ATC = automatic tube compensation; f/VT = frequency to tidal volume ratio; MAP = mean arterial blood pressure; PaCO_2 _= partial carbon dioxide tension in arterial blood; PaO_2_/FiO_2 _= ratio of partial oxygen tension in arterial blood to fraction of inspired oxygen; PSV = pressure support ventilation; SBT = spontaneous breathing trial

### Patient course and outcome

**Figure 1:**
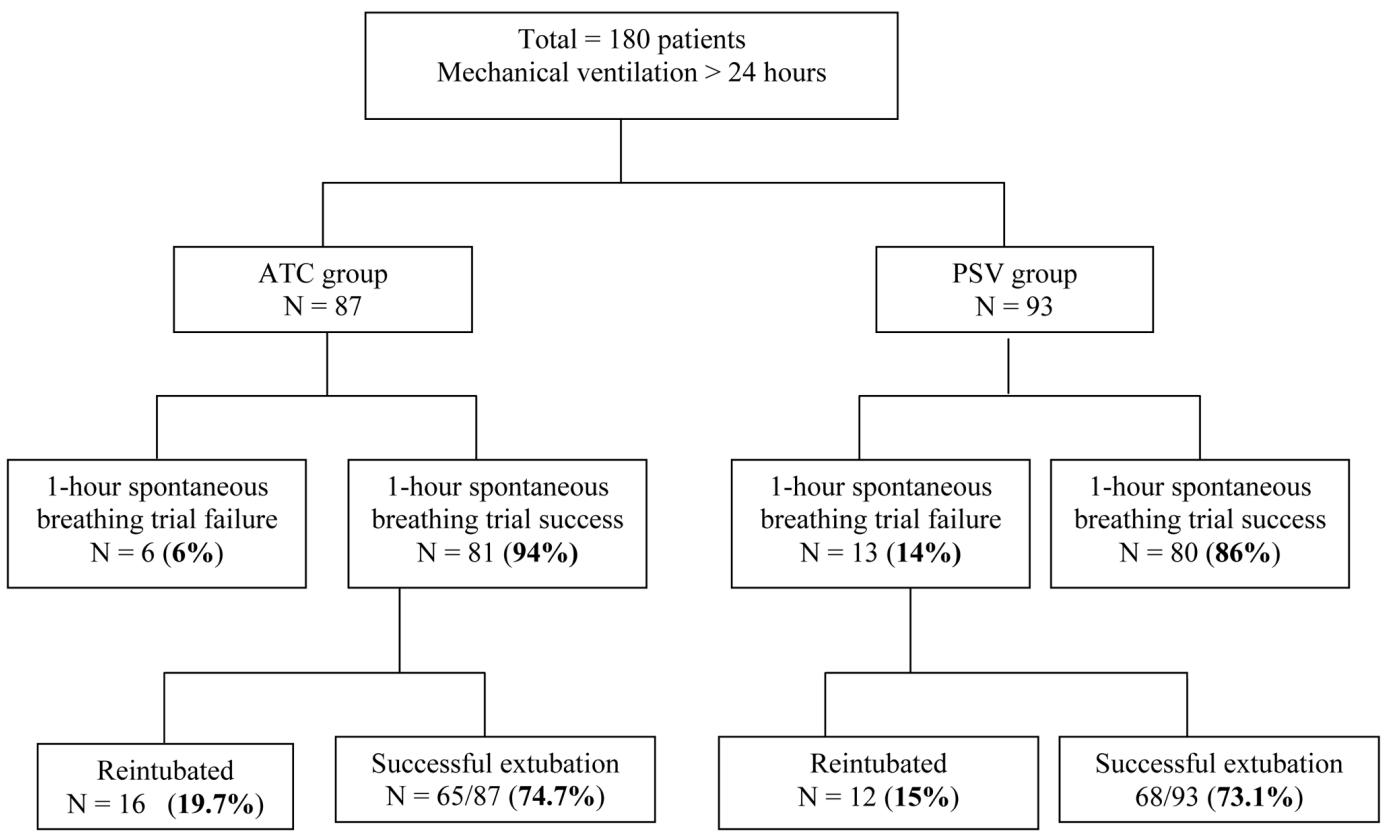
Extubation outcome in the two groups. Automatic tube compensation (ATC) vs. pressure support ventilation (PSV).

These results are shown in Figure 1. In the ATC group, 81 of 87 (93%) patients tolerated the breathing trial and underwent extubation, compared with 80 of 93 (86%) in the PSV group; this observed 8% difference, however, was not significant (p = 0.12). A total of 28 patients (17.3%) required reintubation: 16 (18.4%) in the ATC group and 12 (12.9%) in the PSV group (p = 0.43). Mean time to reintubation was 16.6 hours in the ATC group and 12.8 hours in the PSV group (p = 0.47). Reasons for reintubation were similar in both groups and included hypoxaemia due to inability to clear secretions (n = 16), new sepsis (n = 4), stridor (n = 2), carbon dioxide retention with altered mental status (n = 4) and diaphoresis due to fatigue (n = 2). There was no significant difference between the two groups in the number of patients who remained extubated after 48 hours (ATC, 65 of 87 (74.7%) vs. PSV, 68 of 93 (73.1%); p = 0.81). There was no significant difference in the positive predictive value for successful extubation between PSV and ATC (PSV, 0.85 vs. ATC, 0.80; p = 0.87). No significant differences were noted in patient course or outcome between patients receiving ventilation with either the Evita-4 or Puritan-Bennett 840 ventilators.

### Prediction of extubation by pretest frequency/tidal volume ratio

The results are shown in Table 3. The pretest ATC-assisted f/VT was to found to have a significant contribution in predicting successful liberation and extubation compared with the unassisted f/VT (unassisted f/VT p = 0.19; ATC-assisted f/VT p = 0.005). In ROC analysis with successful liberation and extubation as outcome, the AUC for the ATC-assisted f/VT was 0.70 (standard error 0.083, 95% confidence interval 0.53 to 0.86). The rate of failure of extubation increased from 8.9% for a value of 50 to 75 breaths/minute/L to 24.2% for a value more than 75 breaths/minute/L.

**Table 3:** Results of unassisted and ATC-assisted f/VT in predicting successful extubation outcome

Characteristic	Value	p value
Unassisted f/VT (breaths/minute/L)		
- successful outcome	49.8 ± 22.6	0.19
- unsuccessful outcome	57.0 ± 23.4	
ATC-assisted f/VT (breaths/minute/L)		
- successful outcome	51.4 ± 23.1	0.005
- unsuccessful outcome	69.7 ± 29.6	

Data are presented as mean ± standard deviation.

ATC = automatic tube compensation; f/VT = frequency to tidal volume ratio.

## Discussion

In this prospective, randomised, controlled study, we have shown that the use of ATC during a spontaneous breathing trial was at least as effective as PSV in predicting patients able to maintain spontaneous, unassisted breathing for more than 48 hours after removal of the endotracheal tube and significantly improved the predictive value of the f/VT.

Previous studies have suggested that some level of respiratory support may be beneficial during a spontaneous breathing trial to avoid 'iatrogenic' weaning failure, that is, weaning failure due to the increased work of breathing imposed by the artificial airways. Esteban and colleagues compared extubation outcome after a spontaneous breathing trial with either a T-tube system or low-level PSV (7 cmH_2_O) [14]. They showed that a significantly higher percentage of patients in the PSV group successfully tolerated the trial and underwent extubation (14 vs. 22%, p = 0.03). In a more recent paper, we compared extubation outcome after a spontaneous breathing trial using 100% ATC with CPAP (a supported breathing trial) versus CPAP alone (non-supported breathing trial) in a randomised, prospective study of adult patients in a general ICU [15]. We showed that there was a trend for more patients in the ATC group to tolerate the breathing trial (96% vs. 85%; p = 0.08) although the reintubation rate was similar in the two groups (ATC, 14% vs. CPAP, 24%; p = 0.28). Overall, significantly more patients in the ATC group met criteria for successful extubation, that is, the ability to maintain spontaneous breathing for more than 48 hours after extubation (ATC, 82% vs. CPAP, 65%; p = 0.04).

In the single previous study comparing ATC with PSV during a spontaneous breathing trial, the authors found no significant differences in extubation outcome between the two groups [16]. The authors did find, however, that half the patients who failed a breathing trial with PSV or T-tube tolerated a subsequent trial with ATC and were successfully extubated. The authors concluded that ATC could be used as an alternative mode during the final phase of weaning from mechanical ventilation but that further studies were required. It should be noted that in this study, there were small numbers in each group (n = 30) and the authors used prototype ATC software which is not available with commercial ventilators.

In the present study, we included significantly more patients in each group (ATC, n = 87 and PSV, n = 93) and used commercially available ATC. The baseline characteristics of the two groups were similar apart from a significantly higher APACHE II score in the ATC group. Despite this, we found that 13 of 93 (14%) patients in the PSV group failed the breathing trial compared with only 6 of 87 (7%) in the ATC group; this observed difference of 8% between the two groups, however, did not reach statistical significance (p = 0.12). The fact that ATC may provide more complete support during the spontaneous breathing trial is supported by the results of a recent study, published in abstract form, in which the authors assessed the accuracy of the compensation provided by PSV and ATC relative to the endotracheal tube-related pressure dissipation [17]. They showed that the difference between the theoretical pressure required to overcome the endotracheal tube resistive properties and the actual pressure delivered by the ventilator was lower, always positive and negligible when ATC was applied during a spontaneous breathing trial when compared with PSV (higher difference and frequently negative).

The reintubation rate for the whole cohort was 17.3%, which is compatible with the recent suggestion that an extubation failure rate of 15 to 20% implies an acceptable balance between performing premature extubation and unnecessarily prolonging mechanical ventilation [18]. In addition, the reasons for reintubation and time to reintubation were similar in the two groups. A concern has been raised that by decreasing the work of breathing, ATC could allow more marginal patients to tolerate a breathing trial who would then develop ventilatory failure after extubation [19]. In the present study, the reintubation rate was 12.9% in the PSV group and 18.4% in the ATC group. Although this represents a relative increase of 50%, this did not reach statistical significance (p = 0.43). Regarding the primary outcome measure, that is, the number of patients able to maintain spontaneous breathing for more than 48 hours, we found no significant difference between the two groups (p = 0.808).

The fact that a significant number of patients who pass the breathing test and are extubated subsequently require reintubation has prompted a continued search for parameters that may be used to supplement the predictive value of the spontaneous breathing trial [2,3]. This remains relevant because reintubation has been associated with significant morbidity and even mortality. Although no index has proven to be highly predictive of weaning, the f/VT, a simple bed-side test not dependent on patient cooperation and effort, has been shown to be most consistently and powerfully predictive of extubation outcomes [19]. Indeed, recent reviews continue to include the f/VT as an integral part of weaning protocols [18]. In addition, a recent study showed that the best predictors of extubation failure included the f/VT, degree of fluid balance 24 hours before extubation and pneumonia as the cause for initiating mechanical ventilation [20]. We hypothesised that the predictive value of the f/VT might be further improved by considering the contribution of the endotracheal tube, and that the addition of ATC would result in a 'resistance-free' f/VT, which might more closely mimic the status after extubation. Indeed in this study, the ATC-assisted f/VT performed at the start of the spontaneous breathing trial was found to have a significant contribution in predicting successful extubation beyond the non-significant contribution of the unassisted f/VT (unassisted f/VT, p = 0.19; vs. ATC f/VT, p = 0.005). As suggested by Frutos-Vivar and colleagues, we divided patients into categories according to f/VT using arbitrary steps of 25 breaths/minute/L [20]. We found that a value of f/VT between 50 and 75 breaths/minute/L was associated with failure of extubation rate of 8.9% while the rate was 24.2% for a value of more than 75 breaths/minute/L. We believe that this lower cut-off value of 75 breaths/minute/L (compared with the generally accepted cut-off for the unassisted f/VT of 105 breaths/minute/L) is due to the support with ATC-assistance. These findings also confirm the results of our previous study regarding the usefulness of the ATC-assisted f/VT. In that study we showed that the ATC-assisted f/VT assessed at the end of a 60-minute spontaneous breathing trial, as suggested by Chatila and colleagues [21], significantly improved the prediction of weaning outcome in a general ICU population compared with the unassisted f/VT [22].

There are limitations of this study which should be mentioned. Firstly, we cannot exclude that the lack of significance between the groups (ATC vs. PSV) regarding tolerance of the spontaneous breathing trial and extubation outcome is related to the sample size. Secondly, we did not assess the impact of the mode of ventilation on other important goals of ICU care, namely ICU length of stay and mortality. Although this is the largest study to date comparing ATC with another mode of mechanical ventilation, we suggest that the results of the study warrant additional trials which would include a larger number of patients and be designed to address these specific limitations.

## Conclusions

In this prospective, randomised study we have shown that the use of ATC was at least as effective as PSV in predicting successful extubation outcome after a spontaneous breathing trial. In addition, the predictive value of the f/VT was significantly enhanced when measured with ATC assistance. The present study further confirms that ATC may be a valuable additional mode for use during the final phase of mechanical ventilation.

## Key messages

• Some patients may not tolerate a spontaneous breathing trial because of the respiratory load imposed by the endotracheal tube.

• ATC overcomes the imposed work of breathing due to artificial airways.

• ATC was as at least as effective as PSV in predicting successful extubation outcome after a spontaneous breathing trial.

• The predictive value of the f/VT was significantly enhanced when measured with ATC assistance.

## Abbreviations

APACHE: Acute Physiology and Chronic Health Evaluation; ATC: automatic tube compensation; CPAP: continuous positive airway pressure; FiO_2_: fraction of inspired oxygen; f/VT: frequency to tidal volume ratio; ICU: intensive care unit; PaCO_2_: partial carbon dioxide tension in arterial blood; PaO_2_: partial oxygen tension in arterial blood; PEEP: positive end expiratory pressure; PSV: pressure support ventilation; ROC: receiver operating curves.

## Competing interests

The authors declare that they have no competing interests.

## Authors' contributions

JC contributed to study design, data collection and analysis, and drafted the manuscript. MS contributed to study design and data collection. EG, BF and SL contributed to data collection and manuscript review. PS contributed to study design and drafted the manuscript.
